# Perceptions of Intrauterine Devices among Women in Tanzania

**DOI:** 10.24248/eahrj.v6i1.687

**Published:** 2022-07

**Authors:** Joyce Ibrahim Muna, Gladys Reuben Mahiti

**Affiliations:** a School of Public Health and Social Sciences, Muhimbili University of Health and Allied Sciences, Dar es Salaam, Tanzania.

## Abstract

**Background:**

Intrauterine devices (IUD) are important for ensuring neither unplanned pregnancies, nor unsafe abortions occur as well as it helps spacing children among couples. Despite its advantages, its utilization is still inadequate. The underlying causes of low IUD use needs further exploration for a better understanding as well as appropriate handling of the gaps.

**Aim of the study:**

To explore the perceptions regarding intrauterine devices among women aged 15 to 49 years and barriers to its utilisation in Kinondoni Municipal Council.

**Methodology:**

The study was a community-based exploratory study adopting qualitative approach. Five wards were randomly selected in Kinondoni. Data were collected through in-depth interviews (IDIs) and focus group discussions (FGDs). Study participants were purposively sampled for both IDIs and FGDs. Ten (10) women were interviewed in IDIs from all selected wards and Thirteen FGDs were conducted. Thematic analysis was done to analyze qualitative data, into codes, sub-themes, and broader themes. Data analysis was done by using QSR NVivo version 14.

**Results:**

The study involved 10 women aged 15 to 49 years as key informants and 13 FGDs were conducted involving two groups of women of reproductive age, five groups who ever used the method, and six groups who never used it. The study identified perceived barriers towards IUD related to cultural perspectives, negative perception, fear of side effects, individual's insecurity, perceived benefits related to baby's health and parental benefits. Challenges identified were lack of proper information that is; misinformation and misguidance from the health workers, and negative influencers which include intimacy and devices’ related factors.

**Conclusion:**

This study demonstrated that women have different perceptions regarding utilisation of IUD. Perceptions were categorized into perceived barriers and perceived benefits regarding utilization of IUD. Awareness and knowledge on importance of modern contraceptives (including IUD) should be raised. Also, healthcare workers should ensure enhancement of IUD utilization through teaching the community in a comprehensive manner.

## BACKGROUND

Family planning refers to a conscious effort by a couple to limit or space the number of children through the use of contraceptive methods.^1^ It is a voluntary adapted way of living and thinking by the couples based on their knowledge, attitude and responsible decisions.^2^ It is noted that family planning is critical for preventing unintended pregnancies and unsafe abortions ultimately contributing to reducing maternal and child mortality.^3^

The modern methods have been demonstrated to be more scientifically compelling at forestalling unwanted pregnancies than the traditional methods.^4^ Globally, in 2019, 49% of women aged 15-49 years (a total of 922 million women) were utilising some type of contraception, which is an increment from 42% (a total of 554 million women) that of 1990. Contraceptive use among women aged 15-49 years in 2019 was over 55% in 37 countries^5^ despite increase of contraception use among women aged 15 to 49 years in sub-Saharan Africa from 13% in 1990 to 29% in 2019.^5^

Modern contraceptive methods include combined oral contraceptives (COCs) or “the pill”, Progestogen-only pills (POPs) or “the mini-pill”, Progestogen only injectables, Monthly injectables or combined injectable contraceptives (CIC), Combined contraceptive patch and combined contraceptive vaginal ring (CVR), Intrauterine device (IUD) and many others.^6^

The intrauterine methods are top tier contraceptives as they are long lasting, convenient, well-liked by users, cost-effective, unobtrusive, reversible, and have failure rates less than 1% per year for perfect and typical use and thus rivaling the efficacy of permanent tubal sterilisation.^7^

Currently, the world is addressing the population control due to proper utilization of resources. In developing countries, overpopulation has been a major concern. Though, contraception availability has been widely scaled up, there is poor acceptance of contraception? methods which has been contributed to either ignorance or fear of its complication.^8^ The United Nations Population Fund (UNFPA) reported that in Tanzania, the contraceptive prevalence rate (CPR) for modern methods among currently married women (aged 15-49) is 32%. In addition, the unmet need for family planning for currently married women aged 15 to 49 is 22%.^9^ Furthermore, IUD use in the general population of currently married, reproductive-aged women was quite low (about 1 percent).^10^ Due to low contraceptive prevalence (32% among all women), short birth intervals have been common for example one in five births occurs within 24 months of the previous birth.^10^

The overall utilisation of long-term contraceptives as in 2019 was 18.1%, and it was reported that 3.2% of women aged 15 to 49 years used injectables, 9.1% implants, and only 1.9% utilised IUD.^11^ Therefore, the underlying causes of low IUD use needs exploration for better understanding so that the issues can be appropriately addressed.

This study aimed at exploring the perception regarding IUD among women aged 15-49 years and barriers to utilisation in Kinondoni Municipal Council (MC).

## METHODS

### Study Design

The study adopted an exploratory design employing qualitative approach. The purpose of using this approach was to explore perception regarding IUD among women aged 15-49 years and barriers to utilization in Kinondoni MC.

### Study Setting

The study was conducted at Kinondoni MC. Kinondoni was selected as a study area as it has a low proportion (1.9%) of IUD utilization among women aged 15-49 years in Dar es Salaam.^12^ The Municipality has a total population of 1,775,049 and an average household size of 4.0 according to the 2012 National Census. Out of the total population, 860,802 are male and 914,247 females.^13^ The study was conducted in five (5) wards at Kinondoni MC which were randomly selected.

### Data Collection

A simple random sampling was used to select five wards out of 20 (Kawe, Mbezi juu, Makumbusho, Makongo and Mzimuni) in Kinondoni MC. Two streets were picked randomly from each of the selected wards. From each street, two in-depth interviews (IDIs) were conducted making a total of 10 IDIs. Before the actual data collection, the tools were pre-tested in Kunduchi ward among women of reproductive age.

Thirteen focus group discussions were conducted which included, two groups of women of reproductive age, five groups who ever used IUD, and six groups those who never used IUD. Both IDIs and FGDs involved women aged 15-49 years. FGD discussants and IDI participants were purposively sampled. With the help of local leaders, the study participants were sampled as we believed they had knowledge of the subject and could provide useful information. To facilitate FGDs, participants were separated into youth and adult groups (i.e., 18–29 and 30–49 years). Each group included 8 – 12 participants. The principle of saturation was applied where there was no new data, no new themes, and no new coding. Moreover, interview questions were structured to facilitate asking multiple participants the same questions, otherwise one would not be able to achieve data saturation, as it would be a constantly moving target.^14^ Interviews were audio-recorded. A research assistant helped in taking notes. Two research assistants with a public health background were trained in the interview guide and focus group guide. They were also introduced to research ethics. The review of data collection tools was done to report any complexity of data collection and were discussed on daily basis. Interviewed women were purposively sampled from the community based on their age (15-49 years), use of modern contraceptives (current use or non-use), and residence (currently living in Kinondoni MC) to reflect the range of women living in the community.

### Data Analysis

Textual data were explored using thematic analysis.^15^ The principle investigator listened to the whole recording before transcribing. Then the first rough draft was transcribed. After that, the transcript was revisited and edited. The transcript was read and re-read to be familiar with entire body of data. Then, the data were organized in a meaningful and systematic way. Coding was used to reduce data into small chunks of meaning. After examining codes some of them clearly fitted together into a sub-theme. Similar sub-themes were fitted together into a broader theme that seemed to say something specific about this research question. Analysis was done by using NVivo qualitative data analysis software; QSR International Pty Ltd. Version 12, 2018.

### Methodological Considerations

Several criteria are used in evaluating trustworthiness: Credibility, transferability, dependability, and confirmability.^16^ In this study, the use of purposive sampling to select participants fulfilled the criteria for participation and ensured credibility. Confidentiality was encouraged and participants agreed not to share the discussion outside the group and this increased credibility. The involvement of more than one researcher in the research process ensured dependability that the interpretations emerged in data through triangulation. Description of the study context, selection criteria, data collection, analysis complemented with quotations to facilitate readers were done to assess the transferability of the findings. Before administering the instruments, there was back translation from Kiswahili to English in order to check the accuracy of translation and to meet the criterion of confirmability.

### Ethical Approval

Ethical approval from the Muhimbili University of Health and Allied Sciences (MUHAS), Research Ethics Committee (REC) was granted for this study (reference number; DA.282/298/01. C/). Permission to carry out the study in Kinondoni MC was obtained from the District Medical Officer. Participants in the study were provided with informed consent after being informed about objectives and rationale of the study. Participants in the study were free to choose whether to participate in the study or not. Information collected from the participants was kept confidential, no names or any personal identity appeared in the study documents.

## RESULTS

### Socio-Demographic Characteristics of the Participants

The study involved 10 women aged 15 – 49 years as key informants where a half of them (50.0%) were aged 15 – 24 years. Seven participants had a primary level of education and the remaining three had secondary level of education and above. Of all participants, 8 were married, and two were not married (Table 1).

**TABLE 1:** Socio-demographic characteristics of the IDI participants (N=10)

Variable	Frequency (n)	Percentage (%)
**Age**		
15 – 24	5	50.0
25 – 34	3	30.0
35 – 49	2	20.0
**Level of education**		
Primary level	7	70.0
Secondary level	3	30.0
**Marital status**		
Married	8	80.0
Not married	2	20.0
**Wards**		
Kawe	2	20.0
Mbezi juu	2	20.0
Makumbusho	2	20.0
Makongo	2	20.0
Mzimuni	2	20.0

The study involved 104 women aged 15 – 49 years as FGD participants where 41.3% of them were aged 25 – 34 years. About two thirds (66.4%) had a primary level of education and the. Almost three quarters (72.1%) were married. Participants were evenly distributed by wards (Table 2).

**TABLE 2:** Socio-Demographic Characteristics of the FGD Participants (N=104)

Variable	Frequency (n)	Percentage (%)
**Age**		
15 – 24	39	37.5
25 – 34	43	41.3
35 – 49	22	21.2
**Level of education**		
Primary level	69	66.4
Secondary level	31	29.8
Illiterate	4	3.8
**Marital status**		
Married	75	72.1
Unmarried	29	27.9
**Wards**		
Kawe	21	20.2
Mbezi juu	21	20.2
Makumbusho	21	20.2
Makongo	21	20.2
Mzimuni	20	19.2

### Perceived Barriers Regarding Utilization of IUDs

The study reported numbers of barriers regarding utilisation of IUDs. These include cultural perspectives, negative perception, fear of side effects, and individual's insecurity.

### Restrictive Norms and Taboos Affect IUD Decision-Making

Norms are the agreed upon expectations and rules by which a culture guides the behavior of its members in any given situation. Taboo is a prohibition of certain behavior that is so strict that violating it results in extreme disgust and even expulsion from the group or society.

Negative perceptions associated with norms and taboos have been identified as the barriers regarding utilization of IUDs in Kinondoni MC. It was reported that men as heads of families are the ones to make decisions regarding number of children and when to have a child. It has also been identified that the threat of conflict and violence discouraged women from using IUDs. Moreover, the general community has been reported to have no clear understanding regarding the use of IUDs. One of the participants from FGD reported that:

*“I always listen to what my husband suggests. These are our norms. I cannot use it without his consent. If he ever finds that I use I will be in trouble. Also, in our community, if you are found you do not have a child people might start thinking that you are infertile”*

(FGD R6-non-IUD use Tandale).

From the users' perspective, the study has identified negative perceptions characterized with misconceptions, myths and negative reactions, and number of sexual partners. The study found that some people perceive IUDs as a disturbing device during sexual intercourse. Study participants have reported that there is a lot of misleading information in the community regarding the use of IUDs. One of the FGD participants reported that:

*“In short, the community, which surrounds us to be honest, we are being misled about contraception since everybody speaks his/her own. Some are saying they are causing dizziness, you will find some are saying you will be over bleeding. Therefore, everyone is having her own understanding in the street. One among ten will advise you to use family planning”*

(FGD R3 Tandale in IUD use).

In addition, one of the key informants reported that:

*“Some are saying that IUDs, if you have the habit of having many partners, it is not allowed. This means, if you're having a husband, you're supposed to settle with him meaning you should not cheat.”*

(IDI-06)

Fear of side effects has been identified as a barrier towards utilization of IUDs. Among the perceived side effects were PID which is the infection of one or more of the upper reproductive organs, including the uterus, fallopian tubes, and ovaries, ectopic pregnancy (a fertilized egg implants and grows outside the main cavity of the uterus), and menstrual bleeding disturbances. One of study participants from the IDI reported that:

*“However, what friends are saying about the IUDs is that; it is interrupting the menstrual circle. This means if she was bleeding monthly, it can happen two times a week, stops, and start next week again. In addition, it can lead to pregnancies outside the uterus”*

(IDI-10).

**TABLE 3:** Summary of Codes, Sub-Themes and Themes

CODE	SUB-THEME	THEME
Listen	Subjective norms	Perceived barriers regarding IUD
Permission		
Trouble		
Taboo		
Norms		
Misconceptions	Myths and negative reactions	
Myths and negative reactions		
Have many sexual partners		
Risks of PID	Fear of side effects	
Ectopic pregnancy		
Health risks		
Menstrual bleeding disturbances		
Ashamed	Individual's insecurity	
Fear		
Scared		
Fear of pain		
Better health to a baby	Baby's health	Perceived benefits of using IUD
Feeding practice		
Health risks		
Avoid unplanned pregnancy	Long term method	
Long time		
Avoid health risk when using IUD		
Family planning	Family support	Acceptance of the method
Support		
Husband supports		
No problem	Positive attitude	
Accurate knowledge		
Good method		
Stays longer		
Economic benefits		
Inaccurate Perception	Negative attitude	Disinterest to use IUD
Husband denial		
Fear of pain		
Poor family support		
Large male genitalia	Sexual factors	
Multiple sexual partners		
Sex pleasure		
Displacement of device		
Counseling	Being misinformed	Lack of proper information
Poorly informed		
Doctor scares	Misguidance from health workers	
No clear information		
Sexual pleasure	Intimacy factors	Negative influencers
Partners’ disapproval		
Uncomfortable feeling		
Fears of device disappearance	Device related factors	

Another participant reported that:

*“Am really scared of the method. I fear using the method, because I heard one of my friends saying it can lead to inflammation of the pelvic region”*

### Perceived benefits of using IUDs

The study also established perceived benefits of using intrauterine devices. The identified benefits reported by study participants were the baby's health and parental benefits. Study participants reported that the use of IUDs allows for child spacing hence gives a chance for healthy growth of child as he/she will be breastfed properly and parents will have more time to take care of the child. One of the participants from FGD reported that:

*“Contraception is to prevent to get pregnant before the baby reaches 2 years and make a baby to grow in a good health”*

**(FGD-R1 Kawe in IUD use).**

Participants also reported that since IUD is a better choice for family planning as it is long-term contraceptive method, it allows parents to have more time for economic activities hence alleviating financial difficulties. A key informant reported that:

*“First, one stays for a very long time. This means it can take 3 or 5 years quite different from the injection, which is after every 3 months and you are supposed to take pills every day. So, the IUDs method is much better as I can work longer and earn more before having another pregnancy”*

(IDI-07).

### Barriers to the Utilization of IUDs Lack of Proper Information

Lack of proper information has been identified as among the barriers towards utilization of IUDs. This factor was found to be associated with being misinformed and poor guidance from health workers. Misinformation about IUD was found to be associated with poor counseling from the health workers regarding IUD, and misleading information among family and community members. One of the participants reported that:

*“On the side of the family members, there are those who disappointed me by saying it is not good and it might have side effects. Many disappointed me and few encouraged me. They were saying usually a woman should get her period on time and if you will not bleed on time perhaps, there will be blood clots in the stomach and later on you can get cervical problem or to get cancer*

(FGD-R1 Kawe not in IUD use).”

Poor guidance associated with scary information and unclear information on the use of IUD among health workers was mentioned to be the problem. Women lack counseling and adequate information from professional health workers. One the IDI participants reported that:

*“They are saying if that one stays longer, it usually gets rusty. Therefore, the Doctors in the streets are scaring us to put IUDs because each speaks her own that is why we are scared to put”*

(IDI-11).

### Negative Influencers

Intimacy and devices' related factors were found to be negatively influencing utilization of IUDs among women aged 15-49 in Kinondoni. Intimacy factors negatively influencing IUD utilization were sexual pleasure, partners’ disapproval, and uncomfortable feeling. Some of the participants reported that their husbands do not approve the issue of family planning. One of FGD participants pointed out that:

*“In short, my husband does not want me to use family planning at all. Therefore, if I will use it, you should know that I am using secretly. He does not want me to use it perhaps due to the stories, which we are hearing from outside and that is why when he is coming inside, he is strictly against that issue”*

(FGD-R3 Kawe not in IUD use).

Some women also reported that IUD removes sexual pleasure during sexual intercourse with their husbands. One of the participants reported that:

*“…..we are losing sexual feelings with our husbands. This means we are not interested with them. If you are touched, it is like you're touched by the devil. This means feelings are completely cut off however, they help to do family planning”*

(FGD-R3 Tandale in IUD us).

As stated earlier another negative influencer was device related factors. These include fears of device disappearance and insertion procedures. The study findings report that some women tend to fear that the implant could travel throughout the body and become lost. One of the participants revealed this as she pointed out that:

*“I cannot use it due to the way it is inserted and the way it comes out and get lost and it is a challenge when removing it. It is better to use other ways only. I am scared of the implants because they are saying it is usually disappearing as well”*

**(FGD-R6 Mwananyamala not in IUD use).**

Some participants dislike the insertion procedure of the device. One of the participants reported that:

*“Another thing that makes people to not use the IUDs, it is how it is inserted, until you sleep so, it has many conditions”*

(FGD-R5 Kigogo in IUD use).

In summary, barriers to IUD utilisation are lack of information associated with misinformation from the community and misguidance from health workers; and negative influencers associated with intimacy factors and device-related factors.

## DISCUSSION

The findings have answered the question exploring perception regarding IUD as they indicate perceived barriers (related to subjective norms, myths and negative reactions, fear of side effects, and individual's insecurity), and perceived benefits (baby's health and long-term method).

There are number of perceived barriers to utilization of IUD identified in the current study. The study reported that men as heads of families do not approve the use of IUD, hence women fear violence which could be the result of them using IUD. Disapproval of the method can be associated with negative perception as indicated that the general community has no clear understanding regarding the use of IUD. These finding are supported by the study done by Dyne ^16^ on the influence of perceptions of community norms on current contraceptive use among men and women in Ethiopia and Kenya, which reported that contraceptive use can be shaped by the interaction between the perceived community norms and an individual's own desires. The study showed use of family planning methods is influenced with community perception which is in line with study that reported negative perception lead to low utilisation of the contraceptive methods.^17^ Negative perception on IUD was also observed in the study done by Gbagbo and Kayi ^18^ on the use and discontinuation of IUD in the Greater Accra Region of Ghana.

Fear of side effects was also reported in the current study as respondents perceived that IUD can cause PID, ectopic pregnancy, and menstrual bleeding imbalance. Similar findings that support the current study have been reported in a systematic review done by Allen et al ^19^ on the interventions for pain with IUD insertion suggested that the perception of IUDs as abortifacients, risks of PID and ectopic pregnancy has led to deterred IUD use. The study done in Iran also supports the current study findings as it revealed unwillingness of women to use IUD. The study reported that women had inaccurate perception towards IUD such as fear of pain, of IUDs being larger than the genitalia, and of sexual dysfunction.^20^

The study also demonstrated perceived benefits of using IUD as pointed out by study participants. It was reported that IUD is a good method for child spacing hence supports proper healthy growth of the baby as the parents will have more time nurturing the baby. These findings are supported by the study done by Todd et al^21^ on awareness and interest in IUD use among HIV-positive women in Cape Town, South Africa as it pointed that women perceived IUD having many advantages including child spacing. The finding implies that the way women or community perceive IUD determines its utilization. This has been revealed in the current study findings as they showed that those who perceived IUD as a good method were using/willing to use the method.

Lack of proper information was mentioned as one of the key challenges hindering utilisation of IUD among women. Poor guidance from health workers and misleading information from family members and community at large, were mentioned to be associated with improper information. Some of the participants heard that IUD can lead to cervical problem and even cancer. These findings are supported by the study done by Chakraborty et al^22^ little is known about providers’ knowledge and perceptions of the IUD in developing countries. Nepal's liberal IUD service provision policies allow the opportunity to explore provider knowledge and perceptions across cadres and sectors. This research contributes to an understanding of providers' IUD perceptions in low-resource environments, and increases evidence for IUD task-sharing and private sector involvement. Methods: A questionnaire was administered to 345 nurses and auxiliary nurse midwives (ANMs on Knowledge and perceptions of the IUD among family planning providers in Nepal as it revealed that among the barriers to utilization of IUD were lack of trained staff, limited provision of IUD services, provider bias against long-acting reversible contraception, and poor counseling skills regarding the IUD's advantages and disadvantages.

Fear of inserting a foreign device and uncomfortable feeling of using IUD were mentioned as barriers. Moreover, husbands' disapproval to any method of family planning was mentioned as a hindering factor. Similar findings were reported by Lyus et al and Harper et al on Challenges in translating evidence to practice, the provision of intrauterine contraception and use of the Mirena levonorgestrel-releasing intrauterine system and Paragard Copper-T IUD (CuT380A) IUDs in nulliparous women respectively. It was deduced that conceptual concerns and fears about having a foreign body placed inside their womb, lack of counseling and adequate information about, IUDs from healthcare providers to enable them make informed decisions, and fear of painful insertion also affected utilization.^23,24^ These findings implies that the stem of these challenges is information. The community is not well informed about the method and people are misguided by the close ones and even the healthcare workers.

## CONCLUSION

Hindrances to modern contraceptive use are genuine, and they discourage numerous women from utilizing family planning. To change negative perception regarding IUD, the Directorate of Policy and Planning under the Ministry of Health and other implementing partners dealing with reproductive health should strengthen campaigns to empower women such as emphasis on their education, encouraging gender balance by changing community attitude towards position/status of women in a household and in a society as a whole. Also, the campaigns to raise awareness on importance of modern contraceptives among males (husbands) should be emphasized by the Ministry of Health and other implementing partners dealing with reproductive health and should go along with those involving women. Moreover, health workers should teach the community modern contraceptives (including IUD) in a comprehensive manner aiming at enhancing acceptance and utilization of modern contraceptives.
